# Selective enhancement of insulin sensitivity in the mature adipocyte is sufficient for systemic metabolic improvements

**DOI:** 10.1038/ncomms8906

**Published:** 2015-08-05

**Authors:** Thomas S. Morley, Jonathan Y. Xia, Philipp E. Scherer

**Affiliations:** 1 Department of Internal Medicine, Touchstone Diabetes Center, The University of Texas Southwestern Medical Center, Dallas, Texas 75390-8549, USA; 2 Department of Cell Biology, The University of Texas Southwestern Medical Center, Dallas, Texas 75390-8549, USA

## Abstract

Dysfunctional adipose tissue represents a hallmark of type 2 diabetes and systemic insulin resistance, characterized by fibrotic deposition of collagens and increased immune cell infiltration within the depots. Here we generate an inducible model of loss of function of the protein phosphatase and tensin homologue (PTEN), a phosphatase critically involved in turning off the insulin signal transduction cascade, to assess the role of enhanced insulin signalling specifically in mature adipocytes. These mice gain more weight on chow diet and short-term as well as long-term high-fat diet exposure. Despite the increase in weight, they retain enhanced insulin sensitivity, show improvements in oral glucose tolerance tests, display reduced adipose tissue inflammation and maintain elevated adiponectin levels. These improvements also lead to reduced hepatic steatosis and enhanced hepatic insulin sensitivity. Prolonging insulin action selectively in the mature adipocyte is therefore sufficient to maintain normal systemic metabolic homeostasis.

## Abstract (web summary)

Insulin resistance in adipose tissue is a hallmark of obesity. Here, the authors generate inducible adipocyte-specific PTEN knockout mice to demonstrate that enhanced insulin sensitivity in adipose tissue is directly linked to improved systemic metabolic homeostasis, despite an increase in fat mass.

## Graphical abstract

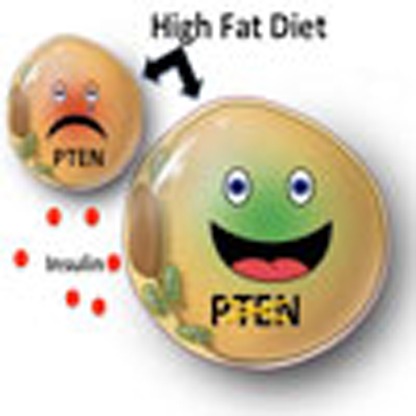

Adipose tissue is an advanced endocrine organ used for the long-term storage of energy-dense lipids^1^. Insulin is primarily responsible for the enhanced uptake of glucose into tissues such as muscle and adipose tissue, suppressing glucagon release from the pancreatic α-cells and concurrently suppressing glucose production and release from hepatocytes. Failure to properly respond to insulin in these respective tissues as a result of insulin resistance triggers elevated glucagon levels and elevated blood glucose levels in peripheral tissues. In the long term, this leads to glucose intolerance and ultimately, type 2 diabetes. While we appreciate the essential nature of the adipocyte as an effective storage cell for excess triglycerides with potent protective action against lipotoxic effects associated with high-fat diet (HFD) exposure, the systemic consequences of prolonged insulin signalling in the mature adipocyte are not well understood. A loss of function of the insulin receptor in adipocytes, the ‘FIRKO’ mouse, displays a lean phenotype and protection against HFD-induced glucose intolerance and lowered fasting triglycerides^2^. A number of additional components of the different branches of the insulin receptor signal transduction cascade have been eliminated from the adipocyte. Adipocyte-specific deletion of Grb10, for instance, leads to inhibition of lipolysis and reduced thermogenesis^3^. Adipocyte-specific loss of Glut4, the insulin-responsive glucose transporter, showed a markedly different phenotype^4^. Using the ap2-CRE mouse to target the *Glut4* gene in adipocytes, Able *et al.* demonstrated significant glucose intolerance and insulin resistance, while adipose mass was preserved. Furthermore, insulin resistance developed in the liver and muscle, secondary to adipose dysfunction. These two observations reflect a dichotomous response to ‘insulin resistance’ in adipose tissue. With the loss of the receptor, we see improvements in systemic glucose tolerance, whereas the loss of a key effector of insulin action, Glut4, results in marked glucose intolerance.

Of interest in this context is the specific role of insulin sensitivity of adipose tissue in orchestrating the synthesis and release of adiponectin. Adiponectin, first identified in 1995 (ref. 5), has potent insulin-sensitizing properties^6^. Indeed, in both humans and mice, adiponectin levels strongly correlate with insulin sensitivity and are generally inversely correlated to body mass index^7^. Although these associations are strong, some of the most insulin-resistant adipose tissues paradoxically produce extremely high levels of adiponectin. In a clinical study, Semple *et al.*^8^ identified a cohort of patients with anti-insulin receptor antibodies that render them extremely insulin resistant; surprisingly, these individuals had markedly increased adiponectin levels in circulation. Furthermore, mice harbouring a knockout of the insulin receptor in adipocytes had elevated intra-adipose tissue levels of adiponectin^2^. In contrast, the vast majority of other models intervening with the canonical insulin signal transduction cascade display lower adiponectin levels. Therefore, with respect to the impact of insulin signalling on adiponectin production, we observe dichotomous consequences, depending on where within the insulin signal transduction cascade the system is tampered with.

In an effort to mechanistically better understand the impact of insulin signalling on adiponectin secretion, we have generated mice that allow us to manipulate insulin sensitivity in adipocytes at the post-receptor level. The phosphatase PTEN (phosphatase and tensin homologue) is a known negative regulator of insulin signalling^9^. We have generated a model that allows us to inducibly delete the PTEN gene specifically in mature adipocytes, thereby greatly prolonging insulin action and enhancing insulin sensitivity, while avoiding any developmental issues associated with the loss of function of this important phosphatase. Using an adiponectin promoter-driven rtTA transgenic mouse, crossed with the tetracycline response element-driven cre-recombinase transgenic mouse (TRE-CRE) in the background of mice carrying a PTEN locus flanked by loxP sites, we can inducibly eliminate PTEN from mature adipocytes upon administration of doxycycline in the feed. This transgenic system has previously been used to track mature adipocytes during embryonic and adult life, and demonstrated to be highly specific and sensitive^10^. Using this model, we can determine the effects of enhanced insulin signalling on adipocytes in adult mice, and can further delineate the impact of prolonged high-level insulin signalling on modulating adiponectin levels in serum and the subsequent impact on whole-body metabolism.

## Results

### Inducible and adipocyte-specific elimination of PTEN

**Figure 1:**
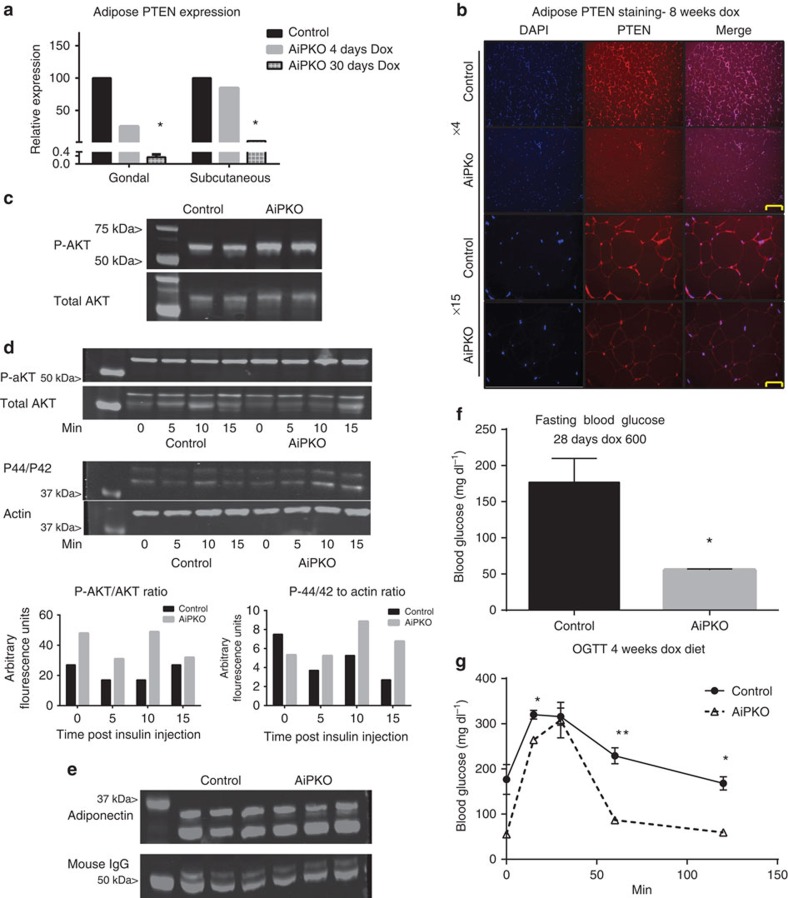
Inducible PTEN elimination from the adipocyte. (**a**) Quantitative PCR results on a floated adipocyte fraction showing PTEN gene expression from mice fed a doxycycline (600 mg kg^−1^) diet for 4 or 30 days (mean of *N*=2 for each bar). (**b**) Gonadal adipose tissue immunofluorescence using a PTEN antibody (Abcam 32199) with 4,6-diamidino-2-phenylindole (DAPI) counterstain to show nuclei. (Scale bar, 152 μm for × 4, 65 μm for × 15.) (**c**) Phospho-AKT, total AKT and actin for isolated adipocytes stimulated with insulin (1 nM) for 10 min (**d**) Western blot of protein extracts from gonadal adipose tissue harvested from mice stimulated with insulin for the indicated amount of time. Staining was then performed against phospho-AKT (S473), total AKT, p44/42 or total actin as indicated and then quantified using a Licor Imager (1 mouse for each group (control or AiPKO) at each time point, 0, 5, 10 or 15 min). (**e**) Western blot for plasma adiponectin levels from mice after 4 weeks of a doxycycline-containing chow diet (*N*=3 for each group). (**f**) Blood glucose of mice fasted for 4 h (*N*=4 for each group). (**g**) Oral glucose tolerance test of mice on a doxycycline-containing chow diet for 4 weeks (average area under the curve—control=28,588, AiPKO=17,001, *P* value=0.0221; Student’s *T*-test) (*N*=3 for each group). All data compare control mice with AiPKO mice. For all points, *P<0.05, **P<0.01 (Student’s *T*-test). All data are mean±s.e.m.

To determine whether the adipocyte-specific inducible PTEN knockout (referred to as AiPKO) mouse is effectively eliminating PTEN expression, AiPKO and control mice were placed on a doxycycline diet (600 mg kg^−1^) for a period of 4 or 30 days. Subcutaneous and gonadal adipose depots were isolated from these mice, fat pads were digested with collagenase and adipocytes were separated from the stromal vascular contents by floatation. Isolated adipocytes were subjected to RNA extraction, and complementary DNA (cDNA) synthesis. Reverse transcription (RT)–quantitative PCR (qPCR) analysis on this cDNA confirmed that after 30 days of exposure to doxycycline, PTEN messenger RNA expression in adipocytes from both subcutaneous and gonadal depots is decreased by over 98% (Fig. 1a). To further confirm these results, adipose tissue was extracted from mice that were fed a doxycycline diet for 8 weeks. Analysis of gonadal adipose tissue by immunofluorescence microscopy confirms widespread loss of PTEN protein from AiPKO adipocytes, while the PTEN signal clearly remained stable in the control adipocytes (Fig. 1b).

As PTEN is a known phosphatase of PiP_3_, a key second messenger in the insulin signalling transduction cascade and an activator of AKT, we wished to determine whether the loss of PTEN had functional consequences in a cell autonomous manner. Adipocytes were isolated from the subcutaneous adipose depot via collagenase digestion and floatation from AiPKO or control mice after 6 weeks of a doxycycline chow diet (600 mg kg^−1^) following a 5-h fast. These adipocytes were stimulated with 1 nM insulin for 10 min, at which time the cells were lysed and proteins extracted for western blot analysis. The AiPKO adipocytes demonstrated enhanced activation of the insulin signalling pathway as judged by increased phosphorylation of AKT (Fig. 1c). To confirm this result *in vivo*, both control and AiPKO mice were placed on a doxycycline diet (600 mg kg^−1^). After 4 weeks, mice were fasted for 4 h, then intraperitoneally injected with insulin (0.5 IU kg^−1^) and euthanised at 0, 5, 10 or 15 min. The adipose tissue from these mice was harvested, proteins were extracted in TNET buffer containing protease and phosphatase inhibitors and then subjected to western blot analysis. This analysis demonstrated enhanced AKT phosphorylation at all time points, as well as enhanced P-42/P-44 activation, indicating enhanced insulin signalling through both pathways of the insulin receptor (Fig. 1d).

To determine whether the observed enhanced insulin signalling in adipose tissue had functional implications for adiponectin secretion, tail vein blood was drawn and western blotting was performed on serum of mice that had been fed a doxycycline-containing chow diet for 4 weeks. Despite the loss of PTEN from the adipocytes, the blot revealed no significant changes in circulating adiponectin levels in the AiPKO mice compared with controls (Fig. 1e). At the time of blood collection, we noted that the AiPKO mice had gained more weight than the control mice over the course of the 4 weeks of doxycycline administration, although these differences were statistically not significant (weight change—control 13.1%, AiPKO 21.0%; *P*=0.082; Student’s *T*-test). Furthermore, during blood collection, serum glucose was measured. AiPKO mice showed markedly lower circulating glucose levels to a degree that these mice suffer from hyperglycaemia (Fig. 1f). To determine whether this lower glucose was pertinent to overall glucose homeostasis, an oral glucose tolerance test (OGTT) was performed on the mice. The AiPKO mice demonstrated significantly better glucose handling compared with the control mice during this challenge (Fig. 1g). These phenotypic changes demonstrating increased weight gain with concomitant lowering of fasted blood glucose and improved glucose tolerance upon a challenge, observed on a standard chow diet, prompted us to test the impact of enhanced metabolic stress on this model.

### AiPKO mice are resistant to HFD insulin resistance

**Figure 2:**
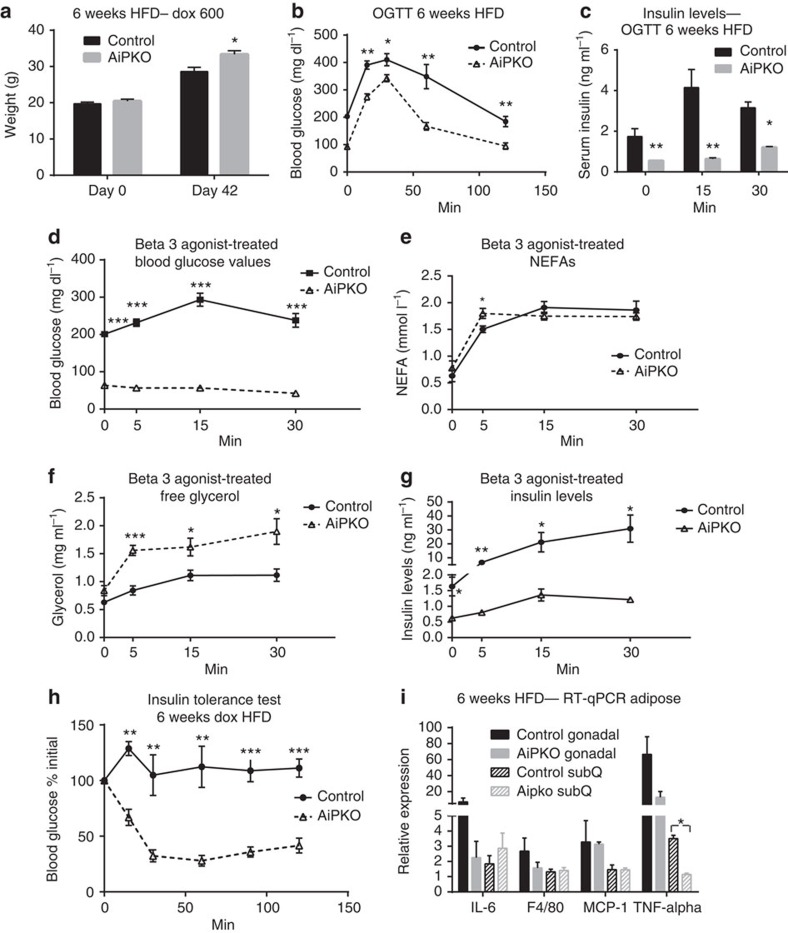
Effects of inducible loss of PTEN in adipocytes during high-fat diet exposure. (**a**) Body weights of mice fed a doxycycline (600 mg kg^−1^) high-fat diet starting at 6 weeks of age for the subsequent 42 days (*N*=5 for each group). (**b**) Concentration of glucose in blood of mice gavaged with 2.5 g kg^−1^ glucose body weight (*n*=5 for each group, average area under the curve—control=37,827, AiPKO=22877, *P*=0.0011). (**c**) Serum insulin concentrations of mice during oral glucose tolerance test shown in (**b**). (**d**) Blood glucose levels in mice following injection of the β3-adrenergic agonist *CL316,243* at 0.5 mg kg^−1^ (*n*=4 control and 5 AiPKO). (**e**) NEFA (**f**) free glycerol and (**g**) insulin levels in the mice from **d**. (**h**) Blood glucose levels displayed as a percent of fasting following injection with insulin (0.5 IU kg^−I^). (**i**) Inflammatory gene expression in adipose tissue from mice fed a HFD doxycycline-containing diet for 6 weeks (*N*=3 for each group). All data compare control mice with AiPKO mice. For all points, *P<0.05, **P<0.01, ***P<0.001 (Student’s *T*-test). All data are mean±s.e.m.

AiPKO mice, starting around 6–7 weeks of age, were switched to a HFD containing 600 mg kg^−1^ doxycycline. After 6 weeks of the HFD challenge, AiPKO mice had gained significantly more weight compared with controls (Fig. 2a). At that time, we subjected the mice to an OGTT. Mice were fasted for 4 h and then gavaged with 2.5 g kg^−1^ glucose (Fig. 2b). The AiPKO mice demonstrated significantly improved glucose tolerance compared with controls, despite the overall weight increase. We also assessed insulin levels over the course of the glucose challenge. AiPKO mice displayed much lower insulin levels compared with their wild-type littermates (Fig. 2c), consistent with an overall improvement in systemic insulin sensitivity.

As the AiPKO mice displayed much lower insulin levels during the glucose challenge, we wished to determine whether this decreased insulin secretion was also observed in the setting of a pharmacologic challenge. Mice were subjected to a β3-adrenergic receptor agonist stimulation (with compound *CL316,243* at 0.5 mg kg^−1^ body weight), injected intraperitoneally following a 4 h fast. This β3-adrenergic receptor stimulation leads to enhanced lipolysis, as well as a rapid and massive release of insulin^11^. Over the course of the experiment, the AiPKO mice displayed reduced glucose levels, resulting in significant hypoglycemia over the entire course of the experiment (Fig. 2d). In contrast, the wild-type mice displayed hyperglycaemia at all times. At every time point, except for the 5-min mark, both wild-type and AiPKO mice overlapped with respect to the increase in lipolysis as judged by significantly increased non-esterified fatty acids (NEFAs) in circulation; in contrast, however, the AipKO mice demonstrated a disproportionately higher level of glycerol release (Fig. 2e,f). Interestingly, the NEFA levels in both genotypes were overlapping, even though the insulin levels in the AIPKO mice were markedly lower compared with wild-type controls (Fig. 2g). In light of the fact that there are no β3-adrenergic receptors present on rodent β-cells that could mediate the insulin release, these results further underscore our previous suggestion that insulin release from the β-cell under these conditions is independent of NEFAs derived from adipocytes, consistent with the release of another secreted factor from adipocytes or a neuronally mediated pathway mediating β3-adrenergic receptor stimulation in adipocytes to prompt insulin release from β-cells.

As these mice demonstrated markedly lower insulin levels during both the OGTT as well as the β3-adrenergic stimulation, we wished to assess whole-body insulin sensitivity after 6 weeks of HFD feeding. Mice were fasted for 4 h and then injected with a submaximal dose of insulin (0.5 IU kg^−1^). Blood glucose was monitored at 15, 30, 60, 90 and 120 min. The AiPKO mice demonstrated a much greater reduction in blood glucose compared with their wild-type littermates (Fig. 2h). These data suggest that these mice are significantly more insulin sensitive, and that alterations of adipose tissue insulin sensitivity is sufficient to completely ameliorate the insulin resistance brought on by HFD feeding.

To determine any changes with respect to adipose tissue homeostasis at the cellular level, gonadal and subcutaneous adipose depots were isolated. QPCR analysis was performed on cDNA samples obtained from these fat pads in an effort to gauge alterations in the inflammatory tone, commonly elevated following HFD exposure. Among the genes tested, tumour necrosis factor alpha (TNFα) was significantly reduced in the subcutaneous adipose tissue of the AiPKO mice compared with wild-type mice, and measurements in gonadal tissue showed similar trends (Fig. 2i). This is a reflection of the overall improved health status of the fat pads in the AipKO mice.

**Figure 3:**
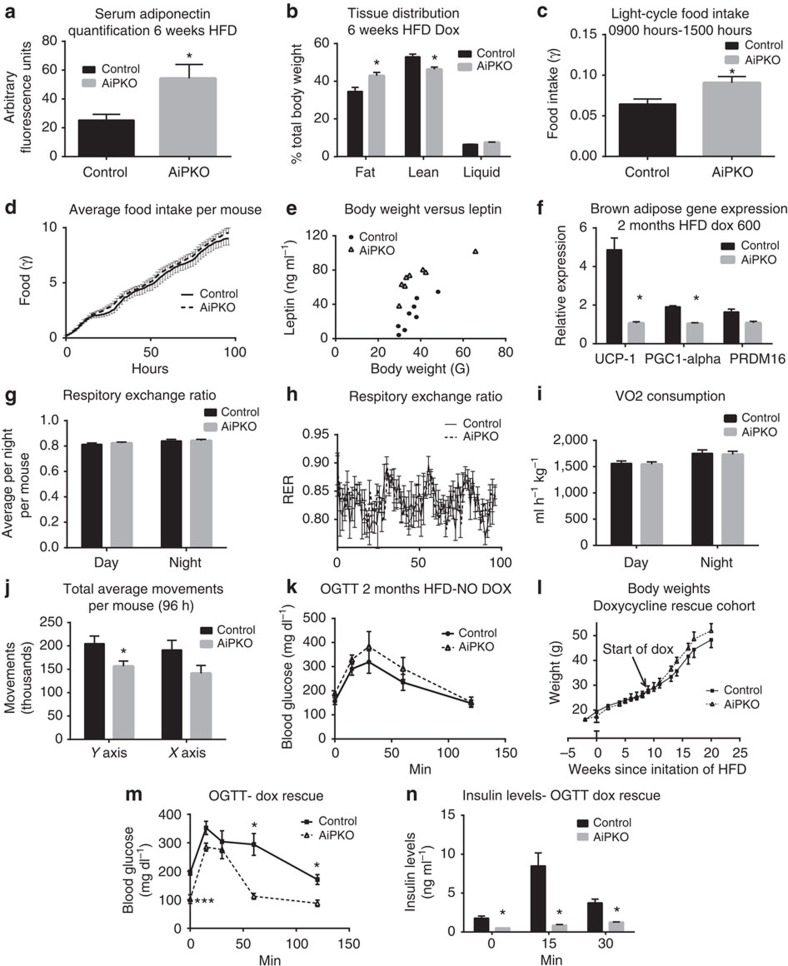
Metabolic characterization after inducible loss of PTEN in adipocytes. (**a**) Serum adiponectin levels of mice fed HFD doxycycline diet for 6 weeks, quantified using a Licor western blot scanner (*N*=6 control and 4 AiPKO). Full blot in Supplementary Fig. 1. (**b**) Tissue distribution determined by a Bruker MQ10 NMR analyzer and expressed as percent of total body weight (*N*=5 in both groups). (**c**) Average food intake per mouse per hour during peak light cycle (sleep cycle, 0900–1500 hours) in a metabolic cage analysis (*N*=4 control and 6 AiPKO). (**d**) Average total food intake per mouse over the 4 days of metabolic cage analysis (*N*=4 control and 6 AiPKOs). (**e**) Circulating leptin levels versus body weight after 6 weeks of HFD containing doxycycline (600 mg kg^−1^) in the AiPKO and control mice as measured by ELISA (Millipore EZML-82K) (*N*=8 for each group). (**f**) Gene expression in brown adipose tissue following 6 weeks of doxycycline (600 mg kg^−1^) containing diet (*N*=3 control and 2 AiPKO). (**g**) Respiratory exchange ratios (RERs) for day and night cycles averaged per 12-h period per mouse (*N*=4 control and 6 AiPKOs). (**h**) Average respiratory exchange ratios for each group taken every 1.5 h over the 4 days of metabolic cage analysis (*N*=4 control and 6 AiPKOs). (**i**) Volume of consumed oxygen for day and night periods, averaged per mouse, per hour, per kilogram (*N*=4 control and 6 AiPKOs). (**j**) Average total movements per mouse during metabolic cage analysis (96 h) (*N*=4 control and 6 AiPKOs). (**k**) Concentration of glucose in blood during OGTT (2.5 g kg^−1^) after 8 weeks of HFD feeding without doxycycline (average AUC; control: 27,725, AiPKO: 32,702, Student's test on AUC *P*-value=0.4246). (**l**) Average body weights of mice in a cohort fed 8 weeks on HFD, followed by 8 more weeks of HFD containing doxycycline (600 mg kg^−1^) (*N*=7 control and 3 AiPKO). (**m**) Concentration of glucose in blood of mice from (**l**) during OGTT (2.5 g kg^−1^) after 8 weeks of HFD feeding without doxycycline followed by 4 weeks of HFD feeding containing doxycycline (600 mg kg^−1^) (average AUC; control: 32,087, AiPKO: 18,951; *P*-value=0.0279). (**n**) Serum insulin concentrations of mice during an oral glucose tolerance test, as shown in (**m**). *P<0.05, **P<0.01. All data are shown as mean±s.e.m.

Since the AiPKO mice demonstrated increased weight gain with improved glucose tolerance following the HFD challenge, we assessed whether this leads to any alterations in circulating adiponectin. Despite the widely established inverse relationship of fat mass with adiponectin levels in circulation, and the generalized increased overall adiposity prompted by the lack of PTEN in the adipocyte, AiPKO mice placed on HFD containing doxycycline for 6 weeks demonstrated significantly higher serum adiponectin compared with controls as judged by western blot analysis of serum (Fig. 3a). Nuclear magnetic resonance (NMR) analysis of these mice revealed an increase in total body adiposity with a concomitant relative decrease in lean body mass (Fig. 3b).

### Metabolic expenditure analysis

In an effort to identify the reasons responsible for the increased fat mass in the AiPKO mice, we subjected a separate cohort to analysis of metabolic chambers. Six-week-old AiPKO mice and their wild-type littermates were put on a doxycycline-containing HFD. After 16 days, mice were placed in metabolic chambers for acclimatization and on day 20 following the initiation of HFD, data collection was initiated. AiPKO mice ate significantly more during the middle of the daytime sleep cycle (from 0900 to 1500 hours on a standard 0600 to 1800 hours light cycle) (Fig. 3c). However, of note is that the overall food intake integrated over the course of the entire 24-h period was not changed (Fig. 3d). As the central hypothalamic axis is responsible for food intake, we sought to determine whether the AiPKO mice had alterations in the anorectic hormone leptin. Serum was collected from mice that had been fed a HFD containing doxycycline (600 mg kg^−1^) for 8 weeks. This serum was analysed by an enzyme-linked immunosorbent assay for leptin and demonstrated that the AiPKO mice had elevated leptin levels compared with the control mice when controlling for body weight (Fig. 3e). These data suggest that although enhanced insulin signalling in adipose tissue may increase leptin secretion, these mice still exhibit leptin resistance.

In an effort to identify other mechanisms of increased weight gain in the AiPKO mice, we looked at other metabolically active tissues. A primary mediator of energy expenditure is the activity of brown adipose tissue (BAT). This thermogenic adipose depot is capable of dissipating a great deal of energy, and we determined whether our mice had any alterations in BAT, as previous reports using the adiponectin-rtTA mouse demonstrated some activity in at least a subset of brown adipocytes^10^. AiPKO and control mice were put on a HFD containing doxycycline (600 mg kg^−1^) for 8 weeks. At this time, the mice were euthanised and RNA was extracted and cDNA was synthesized from the BAT. RT–qPCR analysis of the BAT revealed a significant downregulation of brown adipose-associated genes including *UCP-1*, *PGC1-α* and *PRDM16* in the AiPKO mice compared with controls (Fig. 3f). These data indicate a potential decrease in overall energy expenditure potentially explaining the increased weight gain observed. However, the AiPKO mice did not display any significant alterations in VO_2_, VCO_2_, RER and overall food consumption during the metabolic cage analysis (Fig. 3g–i). Despite the lack of significant alterations in these metabolic parameters, the AiPKO mice moved significantly less than the wild-type littermates in the *y* axis, and showed a trend towards reduced movement in the *x* axis (Fig. 3j). These data suggest that decreased movement and increased food consumption during resting hours are likely to account for the increased overall weight gain seen in AiPKO mice.

### PTEN loss restores insulin sensitivity after HFD exposure

To determine whether loss of PTEN from adipocytes could rescue a pre-existing diabetic phenotype induced by HFD feeding, AiPKO mice and their wild-type littermates were subjected to 8 weeks of HFD feeding starting at 6 weeks of age. At 8 weeks post initiation of HFD, an OGTT revealed no differences between the control mice and the AiPKOs (Fig. 3k). Mice were then switched to a HFD doxycycline-containing diet and were monitored longitudinally. While the weights always trended towards a slight increase in the AiPKO mice after initiation of Dox exposure, these differences never reached statistical significance (Fig. 3l). At 4 weeks following the switch to doxycycline-containing chow, mice were again subjected to an OGTT. The AiPKO mice demonstrated significant improvements compared with their control littermates (Fig. 3m). As before, insulin levels in these AiPKO mice were significantly lower than the controls (Fig. 3n). These observations indicate that the loss of PTEN in the adipocyte, that is, a selective and inducible improvement in adipocytes post exposure to HFD, prompts a reversal of a pre-existing, established diabetic phenotype.

### AiPKO mice after a long-term HFD insult

**Figure 4:**
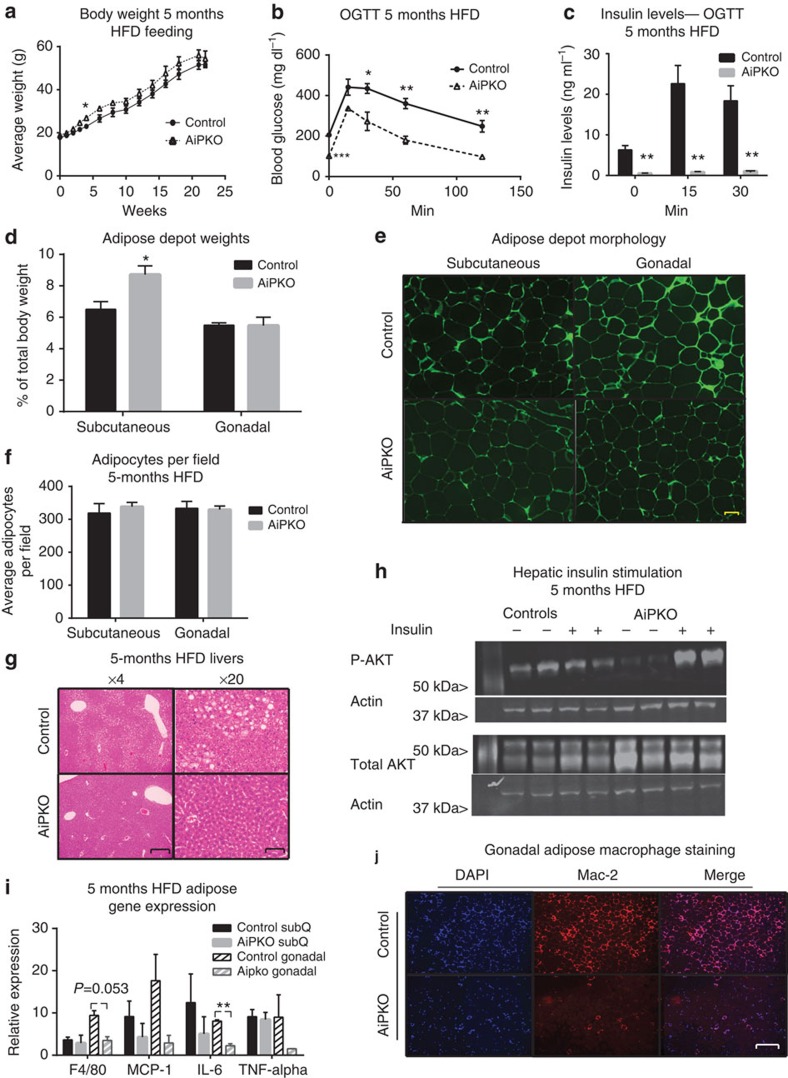
AiPKO mice display improvements even after a long-term HFD insult. (**a**) Body weights of HFD doxycycline- (600 mg kg^−1^) fed mice for 23 weeks (*N*=4 for each group). (**b**) Concentration of glucose in blood of mice gavaged with 2.5 g kg^−1^ body weight (*n*=4 for each group) (average area under the curve—control=41,601, AiPKO=22,960, *P*=0.0007). (**c**) Insulin levels of mice during the oral glucose tolerance test shown in **b**. (**d**) Adipose depot weights at the time of killing for each cohort (*n*=4 for both groups). (**e**) Autofluorescent image (excitation at 480 nm) of adipocytes from AiPKO and control mice (scale bar, 81 μm). (**f**) Quantification of the number of adipocytes observed per field at × 4 magnification using ImageJ software from the subcutaneous and gonadal adipose depots of both control and AiPKO mice (*N*=2 mice for each group (control and AiPKO) and three fields of view for each tissue section, total six fields per group). (**g**) Liver histology (haematoxylin and eosin stain) of control and AiPKO mice after 5 months of HFD doxycycline feeding. Scale bar for the × 4 magnification: 132 μm; for × 20 magnification: 32 μm. (**h**) Western blot of liver protein extracts from mice treated with PBS or insulin for 15 min prior to killing (*N*=4 for each group, 2 receiving insulin and 2 controls). (**i**) Quantitative PCR analysis of adipose depots inflammatory gene expression from controls and AiPKO mice following 5 months of HFD feeding (significance values, subcutaneous adipose—F4/80 *P*=0.7401, MCP-1 *P*=0.4230, Il-6 *P*=0.4475, TNF-α *P*=0.8050, gonadal adipose—F4/80 *P*=0.0538, MCP-1 *P*=0.1506, Il-6 *P*=0.0093, TNF- α *P*=0.2948) (*N*=3 for each group). (**j**) Gonadal adipose depot staining for macrophage infiltration after 5 months of HFD feeding using an anti-Mac2 antibody with a 4,6-diamidino-2-phenylindole (DAPI) counterstain. Scale bar, 132 μm for × 4 magnification. (The experiment was performed on *N*=3 mice for each group.) All data compare control mice with AiPKO mice. For all points, *P<0.05, **P<0.01, ***P value<0.001 (Student’s *T*-test). All data are mean±s.e.m.

To determine whether the metabolic advantages conferred upon the AiPKO mice due to the absence of PTEN were long lasting or whether some resistance to high PTEN starts to appear over time, mice were subjected to prolonged HFD feeding. Body weights of AiPKO mice were significantly elevated after 4 weeks and displayed a trend towards an increase in body weights compared with their control littermates over 24 weeks, although never reaching statistical significance thereafter (Fig. 4a). After 5 months of HFD feeding, AiPKO mice were subject to an OGTT (Fig. 4b). Even after this prolonged metabolic insult, the AiPKO mice handled a glucose bolus much more effectively. It is also apparent that the baseline fasting glucose levels (at the beginning of the OGTT) were much lower than in the control group. Furthermore, insulin levels during the OGTT were a fraction of the levels in the control mice, further illustrating a highly stable phenotype that maintains marked improvements in metabolism even after a prolonged metabolic insult (Fig. 4c). Insulin was injected into these mice after a 4-h fast. Fat pads were then harvested, and their weights were assessed. AiPKO mice had considerably larger subcutaneous fat depots, while the gonadal tissue was unaffected (Fig. 4d). As adipose tissue can expand via hypertrophy or hyperplasia, we analysed sections of both gonadal and subcutaneous adipose sections for cell number (Fig. 4e,f). This analysis revealed similar numbers of cells per field in the sections, regardless of genotype. We conclude therefore that the significant expansion of the subcutaneous adipose depot in the AiPKO mice was due predominantly to an increase in cell number in the tissue, that is, hyperplasia, a distinctive feature of healthy adipose expansion.

In parallel to the experiments above, we determined whether the loss of PTEN in adipose tissue was protective for the development of fatty liver after 5 months of HFD feeding. Histological examination of the liver demonstrated significant fat accumulation in the control mice. This stood in stark contrast to the situation in the AiPKO mice, which lacked fat accumulation in their livers after 5 months of HFD feeding (Fig. 4g). To determine whether this lack of lipid accumulation had functional implications, the livers from the insulin-stimulated mice were also analysed for phospho-AKT levels. AiPKO mice showed significantly greater AKT phosphorylation compared with their wild-type littermate controls (Fig. 4h). Of note is the fact that total AKT levels also increase quite substantially in the AiPKO mice. This demonstrates the significant protective effects on hepatic insulin sensitivity upon selective improvements in insulin sensitivity in adipocytes.

To determine whether the long-term HFD feeding had caused lasting alterations at the cellular level in the adipose tissue, gross structural examination under autofluorescent conditions revealed no change in adipocyte size or number (Fig. 4e). To further confirm any alterations to the adipose tissue, the fat pads of these mice were subjected to RT–qPCR and immunofluorescence analysis. RT–qPCR data show decreased F4/80 expression and significantly less interleukin (IL)-6 production in the gonadal fat depots of AiPKO mice, without significant changes of these immune markers in the subcutaneous depot (Fig. 4i). In contrast to short-term HFD feeding, long-term HFD feeding did not result in statistically significant changes in TNFα expression. To further confirm the increase in macrophage accumulation, immunofluorescent staining for Mac2 in the gonadal fat pad was performed. This analysis demonstrated highly elevated macrophage infiltration in the control mice versus the AiPKO, confirming at the protein level the RT–qPCR analysis (Fig. 4j).

## Discussion

Only one previous manipulation of the insulin receptor cascade leading to an increase in insulin signalling in the adipocyte has been described^12^, and none of the critical constituents have been inducibly eliminated selectively from the mature adipocyte only. In the report by Kurlawalla-Martinez *et al.*, the authors used a constitutive aP2-cre line to eliminate PTEN from adipocytes. Their results differed substantially from ours, and these differences likely stem from two major sources: aP2-driven Cre expression may extend to cell types in other tissues under some circumstances, although this can be controlled for^13^. More importantly, aP2-driven transgene expression can eliminate target genes in early pre-adipocyte progenitors, thereby leading to a developmental phenotype that masks the role of the target protein in the mature adipocyte. The latter of these effects presumably resulted in compensatory developmental adaptations. As a result, these authors failed to see changes in body weight or fasting blood glucose levels, both of which are highly apparent upon elimination of PTEN exclusively from the mature adipocyte, and lead to an underestimation of the relevance of PTEN action in the adipocyte.

To put our observations into additional context, another manuscript on the topic was previously published by Komazawa *et al.*^14^ The phenotype described for their adipocyte-specific PTEN null model (employing a constitutive adiponectin promoter-driven Cre) was fundamentally different from what we describe here; more importantly, these observations were not reproducible, and the paper was subsequently retracted^15^.

The use of inducible transgenic models therefore allows for the evaluation of gene function in the adult mouse and enables a more accurate depiction of the physiological impact of a given protein as compared with congenital loss-of-function approaches that affect the development of tissue. To achieve this in the context of adipose tissue insulin signalling, we used an adiponectin promoter-driven rtTA cassette coupled with a TRE-CRE transgenic mouse to inducibly eliminate a floxed PTEN locus from mature adipocytes. Upon confirming that our inducible model effectively and specifically leads to loss of function of PTEN in adipocytes, we assessed the impact of this alteration on mature adipocytes locally at the cell autonomous level as well as its impact on systemic metabolism. Overall, the loss of PTEN in the mature adipocyte leads to increased insulin sensitivity in multiple peripheral tissues, and a greatly improved metabolic phenotype upon acute and chronic exposure to HFDs. This demonstrates that selective improvements in insulin sensitivity at the level of the adipocyte are sufficient to improve whole-body glucose homeostasis.

In clinical studies as well as rodent and non-human primate studies, the levels of the circulating protein adiponectin have been tightly correlated with insulin sensitivity^7^. Here, we have the surprising finding that animals with highly insulin-sensitive adipose tissue lack a corresponding increase in circulating adiponectin under basal conditions. As the adipose tissue in the AiPKO mice demonstrates increased insulin sensitivity in both arms of the insulin response pathway following HFD challenge, not only the PI3-AKT branch, we argue that insulin sensitivity in adipose tissue *per se* is not sufficient to mediate the regulation of circulating adiponectin (Fig. 1d). These data are supported by clinical studies demonstrating that subjects producing endogenous anti-insulin receptor antibodies display massively elevated circulating adiponectin despite a very high level of insulin resistance^8^. Many questions remain as to how the release of adiponectin is regulated by local insulin sensitivity, but a number of studies hint at mitochondrial metabolism playing a major role in determining how much adiponectin is produced and released from the adipocyte^16^.

Upon challenging our mice with a HFD, a condition that caused the AiPKO mice to gain significantly more weight, we saw increased circulating adiponectin relative to the control mice. With their greater percent body fat and increased insulin sensitivity, these mice do not display the drastic decrease in circulating adiponectin commonly seen in insulin-resistant states. This observation is highly reminiscent of the clinical setting in which patients exposed to the antidiabetic drug thiazolidinediones have significantly elevated adiponectin levels despite increase fat mass^17^. An alternative explanation is that rather than displaying increased adiponectin production on HFD, the AiPKO mice may simply be resistant to the decrease in production commonly seen in wild-type mice. Therefore, circulating adiponectin may be more reliably used as a marker of insulin resistance, rather than obesity.

Selective activation of β3-adrenergic receptors with the agonist *CL316,243* is known to acutely induce adipose tissue lipolysis, increase circulating free fatty acids and insulin. An increase in circulating FFAs can cause a direct activation of GPR40 on β-cells, inducing granule exocytosis and increasing circulating insulin levels in the mouse^11^. We injected a low dose of the selective β3-agonist to the AiPKO mice and the controls. Although the circulating glycerol levels are significantly greater in AiPKO mice following stimulation, the NEFA levels are only mildly elevated at 5 min and equal at all subsequent time points. Therefore, if insulin release is triggered by NEFAs, equal amounts of insulin are expected to be released from the β-cells. However, this is not the case. Despite similar levels of circulating NEFAs in AiPKOs following β3-stimulation, their insulin levels are markedly lower than what is seen in wild-type controls. This brings to bear strong disconnect in the current model of β3-agonist-induced insulin secretion. It remains unclear at this time what the mechanism of β3-agonist-induced insulin secretion is. There are no β3-adrenergic receptors in murine β-cells, so this has to be an effect mediated by receptor activation at the level of the white adipocyte^18^. Yet, the results shown here rule out a direct effect of NEFAs.

Chronic HFD feeding is highly associated with inflammatory adipose tissue^19^. This inflammatory response is most notably dominated by increased macrophage infiltration and concomitant increases in local TNFα and IL-6 levels^20^. Previous reports have demonstrated that systemic inhibition of this inflammatory response by genetic means can improve the metabolic homeostasis of animals^20^. The AiPKO model demonstrates that upon maintaining adipose insulin sensitivity during HFD feeding, there is a marked reduction of the inflammatory response commonly observed under these conditions. Upon a short-term HFD challenge (6 weeks), we see a significant reduction in TNFα secretion in the adipose depot, specifically in the subcutaneous depot. Following long-term HFD feeding (5 months), we see a significant reduction in the maladaptive inflammatory response in the gonadal depot as indicated by decreased overall expression of the macrophage marker F4/80, decreased IL-6 production and decreased crown-like structure staining. These observations indicate that the maladaptive inflammatory response to HFD feeding is dictated, directly or indirectly, by adipose tissue insulin sensitivity. The improved local insulin sensitivity is potently cytoprotective for adipocytes, leading to reduced necrotic adipocyte cell death. This in turn leads to the reduced infiltration of macrophages and long-term improvements in insulin sensitivity. Further, decreases in adipose tissue inflammation are known to dictate inflammatory responses in other metabolically active tissues^21^. Therefore, these findings represent a potential mechanism by which maintenance of insulin signalling in adipose tissue may reduce inflammation at distant sites.

Not unexpectedly, maintaining healthy adipose tissue function in AiPKO mice leads to potent liver-protective effects during HFD exposure. Adiponectin with its potent hepatoprotective actions is likely to be at least partially responsible for this phenomenon^6^. Following 5 months of HFD feeding, the AiPKO livers display significantly less hepatic steatosis compared with controls. Furthermore, the livers are exquisitely sensitive to insulin stimulation as judged by increased phospho-AKT staining following intraperitoneal insulin injection. This model represents an additional case of adipose tissue as a primary driver of whole-body insulin sensitivity and metabolism. Many examples from our own work as well as work from others have illustrated this point, such as the observations by Kahn *et al.* who knocked out the glucose transporter Glut4 specifically from adipocytes, leading to profound systemic disturbances^4^,^22^.

The model presented here is also in support of the concept that expansion of adipose tissue mass (that is, obesity) is in fact a defensive mechanism against caloric overload. We see here that protection from diabetes during caloric overload leads to expansion of the subcutaneous depot, commonly referred to as ‘healthy’ fat expansion. As previous studies in our laboratory have shown, expansion of adipose tissue can have potent protective effects from the metabolic derangements seen during HFD-induced insulin resistance^14^,^19^. Interestingly, in the AiPKO mice, already healthy insulin-sensitive adipose tissue expands greatly in the subcutaneous depot as opposed to the gonadal depot. This is another example of the potent anti-lipotoxic effects that subcutaneous depots can confer upon the system upon ‘healthy’ expansion.

The neutralization of excess calories by effectively storing them in adipose tissue is a central aspect of the anti-lipotoxic actions of adipose tissue expansion. Maintaining a high level of insulin sensitivity in the adipocyte is a crucial ingredient in the complex set of reactions that lead to the ‘healthy’ expansion of adipose tissue that mediates the potent insulin-sensitizing effects of the process. Keeping insulin sensitivity at a high level keeps adipose tissue properly vascularized, maintains an adequate number of adipocytes and keeps the level of extracellular fibrosis at bay. The selective loss of PTEN at the level of the mature adipocyte is sufficient for this process.

## Methods

### Animals

Animal care and experimental protocols were approved by the Institutional Animal Care and Use Committee of the University of Texas Southwestern Medical Center. Adiponectin-rtTA mice were produced as previously described^10^. Tre-Cre (Stock number: 006234) and PTEN-floxed mice (Stock number: 006440) were purchased from Jackson laboratories. All experiments were done using male mice with littermate controls that were adiponectinP-rtTA or TRE-cre negative. Mice were fed a standard chow diet (number 5058, LabDiet), a Dox-chow diet (600 mg kg^−1^ Dox; Bio-Serv), a 60% HFD (D12492, Research Diets Inc.) or a Dox-HFD (600 mg kg^−1^ Dox; Bio-Serv). Following weaning, mice were randomly placed into cages for the remainder of their life before genotyping was performed. No other randomization was used while conducting experiments. No animals were excluded from any experiments, unless they displayed obvious wounds from fighting as determined by our veterinarians.

### Statistical analysis

All animal studies were designed to minimize and control for confounding variables such as mouse strain, gender, age, time of day, fasted/fed state, diet and light cycle. On the basis of previous studies using animal models for metabolic research, we can use as few as six animals per treatment group to achieve statistical power to detect significant differences when measuring RNA, protein and blood metabolites. With the use of inducible mouse models, group sizes were increased to 10 to account for any potential variation imposed by the use of ingested gene induction agents. Metabolic cage studies were performed by a technician who was blinded to the genotype of the mice. For all other animal work, researchers were not blinded to the genotype.

UT Southwestern Medical Center has a site license for the use of GraphPad Prism software (version 6, GraphPad, Inc., San Diego, CA). All data are depicted as mean±s.e.m. All experiments presented have only two groups, and an unpaired Student’s *T*-test was used for statistical significance. All data compare control mice with AiPKO mice under identical conditions. For all points, *P<0.05, **P<0.01, ***P<0.001.

### Metabolic testing

OGTTs were performed as previously described^16^. Briefly, mice were fasted for 4 h before being a gastric gavage of glucose at 2.5 g kg^−1^ body weight. Glucose concentrations were measured using an oxidase-peroxidase assay (Sigma). Insulin and leptin levels were measured using commercial enzyme-linked immunosorbent assays from Millipore. Systemic insulin sensitivity was tested by performing an insulin tolerance test. Mice were fasted for 4 h, and then injected with 0.5 IU kg^−1^ body weight of Humalin-R (Eli Lilly). Blood from the tail was measured for glucose content using Contour glucometer strips (Bayer) at 0, 15, 30, 60, 90 and 120 min. β3-Adrenergic receptor tests were performed as previously described^16^. Briefly, mice were fasted for 4 h, at which time *CL316,243* was administered intraperitoneally at 0.5 mg kg^−1^ body weight. Blood was collected at 0, 5, 15 and 30 min. Glycerol and NEFA measurements were determined using free glycerol reagent (Sigma) and free fatty acid quantification kits (Wako Diagnostics-NEFA-HR2), respectively.

### Quantitative RT–qPCR

RNA was isolated from tissues frozen on liquid nitrogen by homogenization in Trizol Reagent (Invitrogen), and RNA extraction was performed using an RNAeasy RNA extraction kit (Qiagen). RNA was quantified using a Nanodrop instrument. In all, 800 ng of RNA was used to transcribe cDNA using an Iscript kit (Invitrogen). RT–qPCR primers are listed in Supplementary Table 1. The messenger RNA levels were calculated using the comparative threshold cycle (*C*_t_) method.

### Western blotting

Frozen tissue was homogenized in TNET buffer (50 mM Tris-HCl, pH 7.6, 150 mM NaCl, 5 mM EDTA, 1% Triton X-100 with phosphatase inhibitors and protease inhibitors as described in ref. 23 (Sigma). For adipose tissue samples, tissue was homogenized first in the absence of Triton X-100, the fat layer extracted and Triton X-100 was then added to a final concentration of 1%. Proteins were resolved on 11% Tris-Glycine gels and then transferred to nitrocellulose membranes (Bio-Rad). Adiponectin was detected with a rabbit polyclonal antiserum^5^. pAkt (Ser473, antibody #4060) and total Akt (antibody #2920) (Cell Signaling Technology) were used (1:1,000) for insulin signalling studies. Primary antibodies were detected using a secondary immunoglobulin G labelled with infrared dyes emitting at 700 nm (Li-Cor Bioscience #926–32220) or 800 nm (Li-Cor Bioscience #926–32211) (both at 1:5,000 dilutions) and then visualized on a Li-Cor Odyssey infrared scanner (Li-Cor Bioscience). The scanned data were analysed using Odyssey Version 2.1 software (Li-Cor Bioscience).

### Histology and immunofluorescence

Fat pads and livers were excised and fixed overnight in 10% PBS-buffered formalin and were thereafter stored in 50% ethanol. Tissues were sectioned (5 μm) rehydrated and stained using haematoxylin and eosin, or with primary antibodies to PTEN (Abcam 32199; dilution 1:25) or Mac2 (CL8942AP; dilution 1:200, CEDARLANE Laboratories USA Inc.). Slides were mounted using Prolong Gold Antifade reagent with 4,6-diamidino-2-phenylindole (Life Technologies).

### NMR and metabolic cages

Metabolic measurements were obtained continuously using TSE metabolic chambers (TSE Labmaster System, Germany) in an open-circuit indirect calorimetry system. Data were normalized to lean body mass as determined using a Bruker MQ10 NMR analyzer.

### Adipocyte isolation

Adipocytes were isolated for confirmation of gene loss and determination of cell autonomous insulin sensitivity using a collagenase digestion similar to stromal vascular fraction isolation procedures^24^. Briefly, freshly isolated adipose tissue was minced and subjected to 1 h digestion at 37 °C in a PBS-collagenase buffer pH 7.4 (collagenase sigma (10 mg ml^−l^). This digest was centrifuged at 600*g* for 5 min and the floating cells were removed for RNA extraction.

## Additional information

**How to cite this article:** Morley, T. S. *et al.* Selective enhancement of insulin sensitivity in the mature adipocyte is sufficient for systemic metabolic improvements. *Nat. Commun.* 6:7906 doi: 10.1038/ncomms8906 (2015).

## Supplementary Material

Supplementary Information Supplementary Figure 1 and Supplementary Table 1
