# Supplementary material for: Selective enhancement of insulin sensitivity in the mature adipocyte is sufficient for systemic metabolic improvements
Source: Nat Commun. 2015 Aug 5;6:7906. doi: 10.1038/ncomms8906 (PMC4527086; doi:10.1038/ncomms8906)
Supplement: Supplementary Information — Supplementary Figure 1 and Supplementary Table 1 [file ncomms8906-s1.pdf]

Supplementary Figure 1

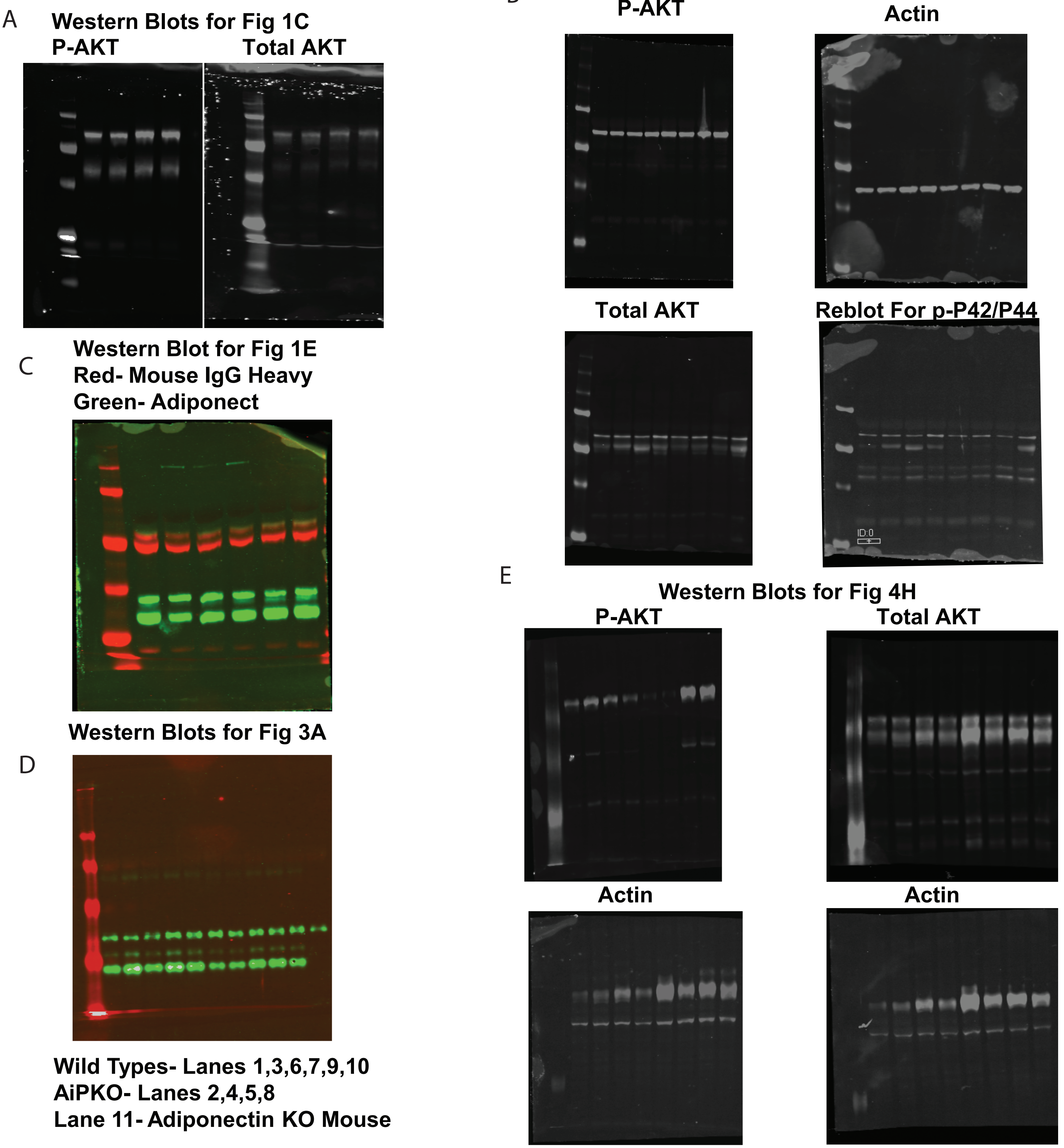

Supplementary Figure 1

Complete set of western blots for all experiments. **A)** Western Blots of P-AKT and total AKT on isolated adipocytes stimulated with 1nm insulin for 10 minutes. **B)** Western Blots of p-AKT and, p-P44/P42, total AKT and actin on isolated gonadal adipose tissue following insulin stimulation for the indicated amout of time. **C)** Western blot of adiponectin and mouse IgG on mice which had been on doxycycline containing chow diet for 4 weeks. **D)** Western blot for adiponectin on serum from mice which had been in HFD food containing doxycycline for 6 weeks **E)** Western blot of P-AKT, AKT and actin on liver protein lysates of mice which had been on HFD for 5 months and were then stimulated with insulin for 15 minutes before sacrifice.

Supplementary Table 1

B-actin	5′-TGGCATTGTTACCAACTGGG	5′-GGGTCATCTTTTCACGGTTG
TNF-α	5′-TCTGTCTACTGAACTTCGGG	5′-ATCTGAGTGTGAGGGTCTG
IL-6	5′-TCCAGTTGCCTTCTTGGGAC	5′-GTACTCCAGAAGACCAGAGG
MCP-1	5′-TAAAAACCTGGATCGGAACCAAA	5′-GCATTAGCTTCAGATTTACGGGT
F4/80	5′-CTCAGTCTGCACCAATATCCTG	5′-CCACAGAGTTAGAGCAGTTGGAA
PRDM16	5′-ACACGCAGTTCTCCAACCTGT	5′-CCCACAGTCCTTGCACTTGAT
UCP-1	5′-TCTCAGCCGGCTTAATGACTG	5′-GGCTTGCATTCTGACCTTCAC
PGC1−α	5′-GCACCAGAAAACAGCTCCAAG	5′- CGTCAAACACAGCTTGACAGC
PTEN	5′-CAATCATGTTGCAGCAATTCACT	5′-CCCCATAAAAATCTAGGGCCTCT

Supplementary Table 1

Quantitative PCR primers used in the studies reported here.
